# Supplementary material for: Exploring issues surrounding mental health and wellbeing across two continents: A preliminary cross-sectional collaborative study between the University of California, Davis, and University of Pretoria
Source: PLoS One. 2020 Oct 23;15(10):e0241302. doi: 10.1371/journal.pone.0241302 (PMC7584185; doi:10.1371/journal.pone.0241302)
Supplement: Attachment — Submitted filename: Response to Reviewers.docx [file pone.0241302.s001.docx]

**31 July 2020**

Dr. Annunziato

Academic Editor- PLOS ONE

Dear Dr., Annunziato,

We are pleased to submit the revised version of the manuscript entitled:

“**A preliminary cross-sectional collaborative study between the University of California, Davis, and University of Pretoria**” to PLOS ONE.

Thank you for giving us the opportunity to revise and resubmit this manuscript. We appreciate the time the reviewer spent on this manuscript and the detailed comments that they have provided. The manuscript has been revised to reflect your suggestions and the reviewer’s comments and suggestions.

All authors have approved the final version of the revised manuscript.

We have responded specifically to each comment/suggestion below and highlighted any changes made to the manuscript in green (‘Manuscript with track changes’).

**Academic Editor’s comments**

I received extensive feedback and I have carefully reviewed the manuscript as well. Overall, we thought that this manuscript presented an important, understudied topic that may generalize more broadly. Our reviewer offered some points of clarification and suggestions for edits that I would ask you to please address in a revision. I also suggest clarifying the statistical analyses and the choice to run univariate followed by multivariate tests. It seems like solely using a multivariate approach like MANOVA would be more appropriate for reducing the number of comparisons and capturing the likely correlations between subscales.

***AU Response:*** *Thank you for the suggestion. We agree that analyzing the data using MANOVA is more appropriate compared to our previous approach that included adjustment of the P-values in the multiple comparisons.*

*We re-analyzed the data using a one-way MANOVA. As a result of performing MANOVA, the following summarizes the changes (or no changes) in the results/conclusions:*

*1. Gender was not a significant predictor of any of the survey instrument scores in this revised version of the manuscript. In our first submission, gender was a significant predictor for emotional exhaustion (for UP students only because only 1 male student completed the survey at UCD) because we had analyzed the data as separate multiple regression models. Similar to our first submission, marital status and age were not significant predictors of scores on the survey instruments.*

*2. Group (UCD or UP) was a significant predictor of scores in at least one survey instrument, consistent with our first submission results/conclusions. Emotional exhaustion scores were higher for UCD students compared to UP students. This conclusion is similar to our first submission results. The P-value was different but still significant (P<0.0001, in our first submission versus P = 0.0023 in this revised version). Scores for depersonalization (P =0.931) and personal accomplishment (P = 0.123) were not different between the two schools, consistent with our first submission results.*

*3. Conclusions for differences between PHQ-9 and GAD-7 scores between UCD and UP remained unchanged, but the P-values changed, though still not significant.*

*4. For SF-8: Social Functioning dimension score which was significantly different between the 2 schools was not significant (P = 0.179) with MANOVA. In contrast, Bodily Pain dimension (previously non-significant in the first submission) was significantly different (P=0.020) in this revised version.*

*The Physical Functioning (P = 0.039) was still significantly different between the two schools, consistent with the conclusion in the first submission.*

*Although the p-values changed, Role Physical (P = 0.443), General Health (P=0.554), Vitality (P = 0.795), Role Emotion (P = 0.114), Mental Health (P = 0.09), Mental Component Summary (P=0.418), and Physical Component Summary (P =**0.727) were not different between the schools, consistent with the conclusion in our first submission.*

*5. We revised the results, included analysis from MANOVA, and deleted results from multiple regression analysis for each survey instruments from our first submission. Please see results.*

*6. We also included the significant relevant correlations between the subscales. Please see results.*

**PLOS ONE style requirements**

1. Please ensure that your manuscript meets PLOS ONE's style requirements, including those for file naming

***AU Response****: We followed all the style requirements and identified the revised editions of the manuscript as suggested by the journal.*

2. During our internal checks, the in-house editorial staff noted that you conducted research or obtained samples in another country. Please check the relevant national regulations and laws applying to foreign researchers and state whether you obtained the required permits and approvals. Please address this in your ethics statement in both the manuscript and submission information.

***AU Response:*** *We provided a project approval number by the University of Pretoria. Please see Materials and Methods.*

3. Thank you for stating in your Funding Statement:

"The study was funded in part by the University Capacity Development Grant of the Department of Higher Education and Training (DHET) of South Africa."

Please provide an amended statement that declares *all* the funding or sources of support (whether external or internal to your organization) received during this study, as detailed online in our guide for authors at http://journals.plos.org/plosone/s/submit-now.  Please also include the statement “There was no additional external funding received for this study.” in your updated Funding Statement.

Please include your amended Funding Statement within your cover letter. We will change the online submission form on your behalf.

***AU Response:*** *We deleted the funding source statement from the Acknowledgments and included it in our cover letter as suggested. We provided information on all the funding sources and included the statement; “There was no additional external funding received for this study” as suggested. We included the following funding source statement: “The study was funded by the University Capacity Development Grant of the Department of Higher Education and Training (DHET) of South Africa, and the UC Davis Faculty Discretionary Support funds. There was no additional external funding received for this study.”*

4. We note that you have indicated that data from this study are available upon request. PLOS only allows data to be available upon request if there are legal or ethical restrictions on sharing data publicly. For information on unacceptable data access restrictions, please see http://journals.plos.org/plosone/s/data-availability#loc-unacceptable-data-access-restrictions.

In your revised cover letter, please address the following prompts:

a) If there are ethical or legal restrictions on sharing a de-identified data set, please explain them in detail (e.g., data contain potentially identifying or sensitive patient information) and who has imposed them (e.g., an ethics committee). Please also provide contact information for a data access committee, ethics committee, or other institutional body to which data requests may be sent.

***AU response****:* *The data presented in our study potentially contain sensitive identifying personal information such as, mental health data which have psychological risks to the respondents. We have provided contacts (non-authors of this manuscript) to which data requests maybe send. The contacts are the respective institutional representatives for the ethics committees:*

*University of California Davis:*

*Office of Research*

*IRB Administration*

*1850 Research Park Drive
Davis, CA 95618-6153*

*Phone: (530) 754-7679
Fax****:*** *(530) 754-7894*

*Email: ORExecutiveMgtAsst@ad3.ucdavis.edu*

*University of Pretoria:*

*Deputy Dean, Postgraduate and Research Ethics*

*Faculty of Humanities*

*Email: PGHumanities@up.ac.za*

b) If there are no restrictions, please upload the minimal anonymized data set necessary to replicate your study findings as either Supporting Information files or to a stable, public repository and provide us with the relevant URLs, DOIs, or accession numbers. Please see http://www.bmj.com/content/340/bmj.c181.long for guidelines on how to de-identify and prepare clinical data for publication. For a list of acceptable repositories, please see http://journals.plos.org/plosone/s/data-availability#loc-recommended-repositories.

We will update your Data Availability statement on your behalf to reflect the information you provide.

***AU response:*** *Please see our response above, in 4(a).*

**Reviewer #1**

*General response: Thank you for taking time to review our manuscript. To make it easier for you to recognize the changes we made to the manuscript, we highlighted the changes in green.*

**Comments to the Author**
1. Is the manuscript technically sound, and do the data support the conclusions?
The manuscript must describe a technically sound piece of scientific research with data that supports the conclusions. Experiments must have been conducted rigorously, with appropriate controls, replication, and sample sizes. The conclusions must be drawn appropriately based on the data presented.

Reviewer #1: Yes

***AU response:*** *Thank you.*

2. Has the statistical analysis been performed appropriately and rigorously?

Reviewer #1: Unsure

***AU response:*** *We considered recommendations from the Academic Editor, specifically to consider MANOVA (multivariate analysis of variance) rather than assessing the difference in scores for each survey instruments as single multivariate regression analysis. We clarified this in the materials and methods. We changed the results accordingly.*

3. Have the authors made all data underlying the findings in their manuscript fully available?

The PLOS Data policy requires authors to make all data underlying the findings described in their manuscript fully available without restriction, with rare exception (please refer to the Data Availability Statement in the manuscript PDF file). The data should be provided as part of the manuscript or its supporting information, or deposited to a public repository. For example, in addition to summary statistics, the data points behind means, medians and variance measures should be available. If there are restrictions on publicly sharing data—e.g. participant privacy or use of data from a third party—those must be specified.

Reviewer #1: No

***AU response****: The data presented in our study potentially contain sensitive identifying personal information such as mental health data which have psychological risks for the respondents. We have provided contacts to which data requests maybe sent. The contacts are the respective institutional representatives for the ethics committees:*

*University of California Davis:*

*Office of Research*

*IRB Administration*

*1850 Research Park Drive
Davis, CA 95618-6153*

*Phone: (530) 754-7679
Fax****:*** *(530) 754-7894*

*Email: ORExecutiveMgtAsst@ad3.ucdavis.edu*

*University of Pretoria:*

*Deputy Dean, Postgraduate and Research Ethics*

*Faculty of Humanities*

*Email: PGHumanities@up.ac.za*

4. Is the manuscript presented in an intelligible fashion and written in standard English?
PLOS ONE does not copyedit accepted manuscripts, so the language in submitted articles must be clear, correct, and unambiguous. Any typographical or grammatical errors should be corrected at revision, so please note any specific errors here.

Reviewer #1: No

***AU response:*** *We revised the manuscript and checked for any grammatical errors to the best of our ability.*

5. Review Comments to the Author
Please use the space provided to explain your answers to the questions above. You may also include additional comments for the author, including concerns about dual publication, research ethics, or publication ethics. (Please upload your review as an attachment if it exceeds 20,000 characters)

***Reviewer #1***: This preliminary research study examines and compares the mental health and wellness between veterinary students at the University of California Davis (UCD) and University of Pretoria (UP). Through measurements of anxiety, burnout, depression, and quality of life of students at comparable stages of their veterinary education, the authors found similar concerns for mental health and wellness between both schools. As such, they conclude that the recommended implementations to increase mental health and wellness in North American veterinary schools may be applicable to the University of Pretoria in South Africa. While these results were inconsistent with the authors’ original hypothesis, I expect that further research assessing the different cultural, social, racial and economic risk factors between North American and South African veterinary students will most likely have different implications for the recommendations that would be most effective for South African students.

The major strengths of the paper are contingent on the authors’ extensive knowledge of the topic and the pertinent existing research. The overall paper and conclusion are strengthened by the authors’ explication of their reasoning behind the choices of the data instruments and methods, as well as providing definitions and explanations for concepts that may not be familiar to individuals outside this specialty, such as those discussed in the “Limitations of the study” section (pages 17-18). This is important as it may help increase collaboration and attention to this topic from a cross-disciplinary perspective. Furthermore, it improves readability and reader comprehension. Another strength of the study is their extensive and honest section outlining the limitations of their research study and design and the implications of these for future studies.
However, with this being said, the extensive limitations of the study are what I perceive to be the major weakness of this paper. The various flaws of the study design, including the generalizability and external validity of results, most likely significantly impact the results and conclusions of this study. This may weaken its reproducibility and significance. Other major and minor issues are discussed below.

***AU response:*** *Thank you for your succinct summary of the strength and weaknesses of our study. We agree with your comment that further longitudinal studies results might differ from this current study particularly when cultural, social, racial, and economic factors are considered. While all these factors have a potential influence on the mental health, the degree to which they affect mental health among veterinary students might be different between the schools. We plan to pursue longitudinal studies with intervention strategies at both schools.*

**Major Issues**
1. The authors identify that mental health and wellness research is a topic of importance in North American veterinary schools (line 36). However, they fail to explain why it is important for this specific discipline. With a brief explanation of the importance of this and future research, the authors will provide readers and future researchers with a stronger impetus to perform further research studies, such as those recommended at the end (“Implications of this study for collaborative future studies” section on page 17).

***AU response:*** *We added relevant statistics from recent studies to provide more context on mental health and wellness in North American veterinary students compared to peers in other programs, such as medical students. We added the information in the introduction and cited relevant references. Please see the introduction.*

2. In the Introduction (lines 59-76), the authors identify certain curricular revisions that have been implemented in North American veterinary schools, with specific attention to those at UCD. To strengthen the association the authors make between mental health and wellness in veterinary students with modifications to and change of curriculum, a brief discussion of the success of current curriculum reforms would be helpful. Furthermore, the authors should consider briefly describing the similarities between the curriculum changes implemented at UCD and UP. Failure to describe the curriculum changes that have been implemented at UP is a significant issue that should be addressed to increase the strength of the authors’ conclusion.

***AU response:*** *The revised curricula were implemented in 2011 and 2016, at UCD and UP, respectively. As you pointed out, the revised curricula will have an impact on student mental health and wellness. Curricular evaluation at UCD is planned in 2021 (5 years after the first class on the new curriculum graduated) and 2022 at University of Pretoria. In response to your comment, we added the lack of data regarding the impact of the curricular change on mental and wellness as limitation of our study. Please see changes under ‘Limitations of the study’.*

*While not numerous, we included the similarities between the drivers for curricular changes between UCD and UP. Please see introduction.*

**Minor Issues**

1.While the authors exemplify extensive knowledge of the topic, there are many grammatical errors that make certain sections difficult to read and comprehend, specifically the common occurrence of run-on sentences. Specific attention should be given to the Introduction section.

***AU response****: We revised the introduction and split the run-on sentences where necessary but did our best to maintain flow of the manuscript.*

2. The authors identify the independent variables of the study, which are group, gender, marital status, and age. While they reference previous studies that found significant differences by gender in North America, further explanation for their choice to include marital status and age should be briefly discussed. Additionally, race would have been an easy descriptive statistic to obtain and incorporate into the research. This is an important factor to consider as there is significant evidence of racial disparities in mental health.

***AU response****: As indicated in our first submission, most students from UCD are 25 years and younger (at this stage of the curriculum) and this is expected in North American veterinary schools. Only 1 student was aged 26-30 from UCD 13 students from UP were aged 26-30 hence no comparison was made between the schools. In response to your comment, we did the following:*

*1. Discussed potential reasons why we included marital status and age variables for predicting scores. Please see Discussion.*

*2. We provided descriptive statistics of the racial composition of the 2 classes included in this study. Please see Results.*

3. It is important that the authors included a section on the current mental health and wellness support for veterinary students at UCD and UP. However, positioning this section as part of the Study Design and Methods section is confusing as it is merely background information and not relevant to how the study was conducted. As such, I advise that the authors move this section to be included in the Introduction for coherency and improved flow of the paper.

***AU response:*** *Thank you for the suggestion. We moved the material on mental and health support for UCD and UP to the introduction. We provided sub-titles in the introduction to improve the flow of the manuscript. Please see changes in the introduction.*

4. Although the Discussion section is strong in discussing the results and their implications, it is not coherently structured. It would be helpful too to consider broader implications.

***AU response:*** *We expanded the discussion to include broader implications of the study. Specifically, we discussed the impact of the study results relating to the following:*

*1. The importance of collecting data and monitoring mental health and wellbeing to increase awareness by students and faculty.*

*2. Sharing and provision of resources between schools to monitor mental health and wellness.*

*To improve the flow of the Discussion, we included this information under “Implications of the study for future studies”. We think the information fits better in this part of the discussion as it summarizes the practical implications of the study.*

*Please see Discussion for the additions.*
