# Supplementary material for: Exploring issues surrounding mental health and wellbeing across two continents: A preliminary cross-sectional collaborative study between the University of California, Davis, and University of Pretoria
Source: PLoS One. 2020 Oct 23;15(10):e0241302. doi: 10.1371/journal.pone.0241302 (PMC7584185; doi:10.1371/journal.pone.0241302)
Supplement: Attachment — Submitted filename: Response to Reviewers.docx [file pone.0241302.s002.docx]

Reviewer #1: The authors made many revisions that have significantly strengthened their paper. Aside from the strengths discussed in their first submission, one strong addition was the inclusion of past studies that explicate the importance of studying mental health and wellness in veterinary school students. This will provide a further impetus for future studies to be conducted. The manuscript is further strengthened by the addition of the “Data Analysis” subsection, format changes to improve the paper’s readability, as well as the explanation of the specific independent variables that were studied.

*Response
Thank you for taking time to review our manuscript. We responded point-by-point to your suggestions and comments. To make it easier for you to recognize the changes we made in the manuscript, we highlighted the changes in green where necessary.*

Major Revisions
• The authors state that the factors associated with good mental health and well-being are different between the two countries. They hypothesize that the levels of mental health and wellness would be different between the two schools. However, in the introduction, the authors also hypothesize that the approaches to improve mental health and well-being in North America may be applicable South African veterinary schools. The authors should include a brief explanation as to why they hypothesized this despite the many differences.

*Response*
*We hypothesized that the levels of mental health and wellness would be different based on the different stressors for being in veterinary school between the two different continents. Different stressors that will make approaches to mental health and wellness different between the schools maybe be stressors like powercuts (reducing study time), and isolation of the veterinary school in South Africa (there is only one veterinary school in South Africa); whereas perceived poor physical health and unclear expectations of the curriculum maybe be major stressors at North American schools. However, approaches to improving mental health maybe similar for stressors that are common to being in veterinary school such as heavy academic workload, and lack of racial diversity in class composition.*

*In response to your comment we clarified the statement in the introduction to indicate that although there are different stressors between the schools, improvement of mental health and wellness maybe applicable towards stressors that are common to both veterinary school.*

• The authors state that they are assessing differences between North American and South African veterinary school students. However, all of the data provided, as well as the study conducted, are only applicable to the United States. Therefore, I recommend revising this inaccuracy by replacing “North America(n)” with “the United States of America” or “America(n),” unless the authors can provide statistics that indicate there are comparable mental health and wellness concerns in both Canada and Mexico.

*Response*

*Thank you. We changed the phrase from “North America” to the “United States of America (US)” throughout the manuscript.*

• Throughout the paper, the authors switch between “South Africa(n)” and “Africa(n).”

*Response*

*We changed the phrase to “South African” throughout the manuscript because our study focused on South African students.*

Minor Revisions

• I recommend moving the primary research question stated in lines 141-143 to the abstract.

*Response*

*We added the primary research question to the abstract. To maintain flow of the manuscript, we left the primary research question in the introduction under the sub-title “Current Study” as well.*

• The “Current mental health and wellness support for veterinary students” section is important. While they discuss the UCD “Wellness Center” and the programs it offers, the pathway to UP Counselling Services is less clear. I suggest briefly stating the programs it offers, if the information is available, to demonstrate consistency.

*Response*

*The information indicated in the manuscript for UP Counselling Services is how the program is set-up currently and there is no additional information regarding its organization. Most of the staff administering the program are part-time except the full-time faculty advisor. However, the full-time faculty advisor provides mentoring and life coaching, but is not qualified/licensed to provide psychotherapy. The program at UP is still growing at the moment.*

• To improve flow, readability and comprehension of the “Results” section, I suggest putting each school’s information consecutively. For example, state ages of respondents for UP followed by the ages of respondents for UCD. Or create separate paragraphs for each school.

*Response*
*Thank you. We re-organized the results as suggested. Please see the results.*

• Although very minor, I suggest creating a new paragraph on line 93 to separate the background information discussion of North American and South African veterinary schools.

*Response*

*We created a new paragraph to separate background information on US veterinary schools and the South African school. Please see introduction.*

• The authors made many corrections to grammatical errors present in their first submission. However, there are still many run-on sentences and misuses of commas. I recommend revising these grammatical errors to improve readability and comprehension.

*Response*

*Thank you for the comment. We revised the manuscript and divided the run-on sentences when necessary. We also removed inappropriate commas when necessary using Microsoft Word grammar and spell-check.*
